# Novel Roles of the Nipah Virus Attachment Glycoprotein and Its Mobility in Early and Late Membrane Fusion Steps

**DOI:** 10.1128/mbio.03222-21

**Published:** 2022-05-04

**Authors:** Victoria Ortega, J. Lizbeth Reyes Zamora, I. Abrrey Monreal, Daniel T. Hoffman, Shahrzad Ezzatpour, Gunner P. Johnston, Erik M. Contreras, Fernando J. Vilchez-Delgado, Hector C. Aguilar

**Affiliations:** a Department of Microbiology and Immunology, College of Veterinary Medicine, Cornell University, Ithaca, New York, USA; Washington University in St. Louis School of Medicine; University of Pennsylvania

**Keywords:** attachment, fusion, oligomer, paramyxovirus, receptor binding protein, stalk, virus

## Abstract

The Paramyxoviridae family comprises important pathogens that include measles (MeV), mumps, parainfluenza, and the emerging deadly zoonotic Nipah virus (NiV) and Hendra virus (HeV). Paramyxoviral entry into cells requires viral-cell membrane fusion, and formation of paramyxoviral pathognomonic syncytia requires cell-cell membrane fusion. Both events are coordinated by intricate interactions between the tetrameric attachment (G/H/HN) and trimeric fusion (F) glycoproteins. We report that receptor binding induces conformational changes in NiV G that expose its stalk domain, which triggers F through a cascade from prefusion to prehairpin intermediate (PHI) to postfusion conformations, executing membrane fusion. To decipher how the NiV G stalk may trigger F, we introduced cysteines along the G stalk to increase tetrameric strength and restrict stalk mobility. While most point mutants displayed near-wild-type levels of cell surface expression and receptor binding, most yielded increased NiV G oligomeric strength, and showed remarkably strong defects in syncytium formation. Furthermore, most of these mutants displayed stronger F/G interactions and significant defects in their ability to trigger F, indicating that NiV G stalk mobility is key to proper F triggering via moderate G/F interactions. Also remarkably, a mutant capable of triggering F and of fusion pore formation yielded little syncytium formation, implicating G or G/F interactions in a late step occurring post fusion pore formation, such as the extensive fusion pore expansion required for syncytium formation. This study uncovers novel mechanisms by which the G stalk and its oligomerization/mobility affect G/F interactions, the triggering of F, and a late fusion pore expansion step—exciting novel findings for paramyxoviral attachment glycoproteins.

## INTRODUCTION

Paramyxoviruses are enveloped, negative-sense single-stranded RNA viruses (1). They are generally highly infectious, and their host range includes mammals, fish, birds, and reptiles (2–5). They are medically, agriculturally, and economically important, and comprise measles, mumps, various human parainfluenza viruses, avian paramyxovirus 1 (formerly known as Newcastle disease virus), canine distemper, and the deadly zoonotic henipaviruses Hendra virus (HeV) and Nipah virus (NiV) (1).

A common cytopathic effect of paramyxoviral infections is multinucleated syncytium formation by the fusion of infected host cells with infected or naive cells (6). Viral entry into host cells and syncytium formation are both mediated by viral surface glycoproteins (6). Paramyxoviruses contain two viral surface proteins, namely (i) receptor binding protein (RBP) G, RBP H, or RBP HN and (ii) the fusion (F) glycoproteins. The paramyxoviral RBPs are tetrameric type II transmembrane proteins, while Fs are trimeric type I transmembrane proteins (5, 7). The RBPs consist of four domains, the cytoplasmic tail, transmembrane, extracellular stalk, and extracellular globular head domains. Their designations are based loosely on protein function and include hemagglutinin-neuraminidase (HN), hemagglutinin (H), and glycoprotein (G) (7–10). Henipaviral G glycoproteins have neither hemagglutination nor neuraminidase activities and typically bind the cellular receptors ephrinB2, ephrinB3, other ephrins, and possibly other protein receptors (11–15).

The current knowledge of the process of paramyxoviral membrane fusion and entry into host cells can be summarized as follows: the RBP interacts with the host cell receptor and then undergoes receptor-induced conformational changes that result in the RBP stalk being exposed to the F glycoprotein (16, 17). This conformational change triggers F to initiate its own conformational cascade that progresses from prefusion to prehairpin intermediate (PHI) to postfusion conformations (18, 19). At the PHI conformation, the fusion peptide harpoons itself into the host cell membrane; then, heptad repeat regions 1 (HR1) and 2 (HR2) in the F ectodomain, which have a high affinity for each other, come together to form a 6-helix bundle in the F postfusion conformation. This process brings the host and/or viral lipid bilayers together. First, the outer leaflets intermix in a hemifusion state. Then, the inner leaflets mix to form a full fusion pore, which is then expanded to allow mixing of viral and host cell cytosolic environments (viral entry) (20). In the case of cell-cell fusion, the fusion pore needs to expand extensively to allow the conglomeration of nuclei from multiple and typically numerous cells, forming the syncytia characteristic of paramyxoviral infections (12, 21–24).

For decades, the paramyxoviral RBPs were thought to function only early in the membrane fusion process, such as in binding to the host receptor and in promoting F triggering. However, recent evidence suggests that the HPIV-3 HN may potentially play a role later in the membrane fusion cascade (25, 26). However, the specific role(s) of the RBP and/or the G/F interactions in any specific step(s) of the membrane fusion cascade after F triggering remains unknown.

Here, we demonstrate a novel role of the NiV G stalk and of G/F interactions in the triggering of the F protein, as well as in a specific post-F-triggering step, late fusion pore expansion. These findings challenge past dogma about membrane fusion cascade. These discoveries came about by restricting the movement of specific regions along the NiV G stalk by introducing individual cysteines along the stalk from the N terminus to the C terminus. Therefore, this study offers new understanding of how the mobility of the G stalk domain affects the strength of G/F interactions, the triggering of the fusion protein, and a late membrane fusion step, with potential implications beyond the henipaviruses for other paramyxoviruses and for the ways we can combat their pathogenesis.

## RESULTS

### NiV G stalk cysteine additions increase tetrameric structural strength.

**FIG 1:**
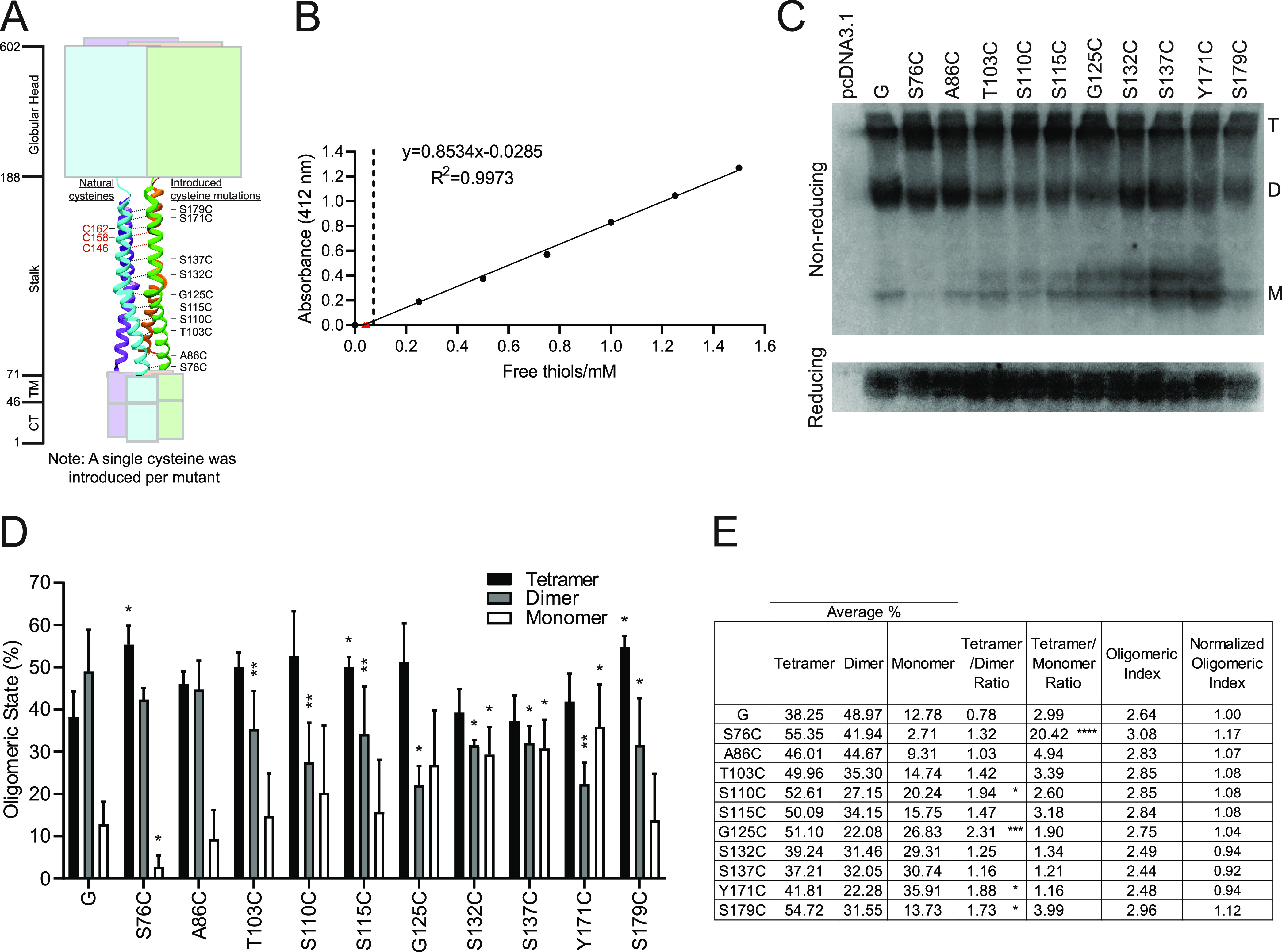
NiV G cysteine mutants modulate oligomeric structure. (A) Representation of monomeric full-length NiV G with all of its domains. CT, cytoplasmic tail; TM, transmembrane domain. The numbers to the left indicate the end of each domain. Drawing adapted from the parainfluenza virus 5 HN glycoprotein (PDB identifier 3TSI) is used to give a visual representation of general cysteine residue mutation location. To the right of the drawing are the amino acids mutated into a cysteine residue. Only one cysteine residue mutation was introduced per mutant. The natural cysteines present in wild-type NiV G stalk are indicated by red text. Image was generated using UCSF Chimera and Adobe Illustrator (69, 70). (B) Determination of no free thiols in wild-type or cysteine mutant NiV G. The dashed black line is placed at the expected concentration of a single reduced free thiol. Red triangles indicate the values obtained for wild-type or cysteine mutant NiV G proteins. These data indicate that there are no free thiols present for any NiV G protein. (C) Representative Western blot analysis of total cell lysates from HEK293T cells transfected with wild-type NiV G or cysteine mutant expression plasmids. (D) Oligomeric states for each of the mutants as tetramers, dimers, or monomers in nonreducing conditions; *N* = 4. (E) Average percentages of tetramers, dimers, and monomers. Tetramer-forming propensity is measured as the following ratios: tetramer/dimer and tetramer/monomer. One-way analysis of variance (ANOVA) was completed for the tetramer/dimer and tetramer/monomer ratios, with significance when *P* < 0.05 indicated with an asterisk (*) and values in bold. Oligomeric index was determined as the sum of the fractions of each of the oligomeric forms (T, tetramer; D, dimer; M, monomer) multiplied by either 4, 2, or 1. Thus, the oligomeric index = (T × 4) + (D × 2) + (M × 1).

We previously determined that the NiV G stalk triggers F (16, 17). To decipher whether the mobility of the various regions(s) of the NiV G stalk domain would be important for F triggering and membrane fusion modulation, we introduced cysteines along the G stalk from the N terminus to the C terminus via site-directed mutagenesis, as shown in Fig. 1A. The goal was to create new Cys-Cys bonds between G monomers, increasing oligomeric (dimeric and/or tetrameric) strength and restricting the mobility of specific regions along the G stalk. A region of the NiV G stalk that already contains three natural cysteines (C146, C158, and C162) that are conserved among henipaviruses such as Hendra, Ghana, and Cedar viruses and reported to be important for the dimeric and/or tetrameric (dimer-of-dimer) G structures (27), as well as its immediately surrounding residues, was left unaltered (Fig. 1A, left, red).

We ran an Ellman’s reagent assay to quantify any potential free thiols present in wild-type or cysteine mutant NiV G (Fig. 1B). These data suggest the protein’s thiols are present in an oxidized state, which indicates that no free thiols have been created and therefore that cysteines within monomers have been paired and disulfide bonded in the G dimers and tetramers. We then transfected the wild-type or mutant NiV G cysteine expression constructs into human embryonic kidney (HEK293T) cells and determined the total cell expression and oligomerization status of each glycoprotein in whole-cell lysates by reducing or nonreducing SDS-PAGE analyses, probing for the extracellular C-terminal hemagglutinin (HA) epitope tag in NiV G, which we have shown does not affect G protein functions (28–30). Like the wild-type NiV G protein, all of the G Cys mutants were expressed well in cell lysates and were able to form monomers, dimers, and tetramers under nonreducing conditions (shown in Fig. 1C and quantified in Fig. 1D). However, as we hypothesized, we observed a shift from a relatively more dimeric to a relatively more tetrameric structural propensity for some of the mutants analyzed (S110, G125, and Y179), while we also observed a relatively more monomeric propensity for other mutants (S132, S137C, and Y171C), or a higher tetramer to monomer ratio for mutant S76C (Fig. 1C, D, E). Notably, the fairly quantitative nature of the fluorescence-based Western blot analysis we used allows us to rely on densitometry over several orders of magnitude of linear range (16, 31, 32). Also notably, most of the stalk mutants that yielded significantly higher tetramer/dimer ratios were at the central and C-terminal regions of the stalk (*P* < 0.01 compared to wild-type NiV G), similarly to what was observed for MeV H (33). To account for all oligomeric forms of G, we also calculated an oligomeric index as the sum of the fraction of tetramers times 4, the fraction of dimers times 2, and the fraction of monomers times 1, and we normalized it to set the oligomeric index for wild-type G as 1.0 (Fig. 1E). We used this oligomeric index for drawing comparisons in subsequent studies.

### Addition of cysteines to the G stalk significantly decreased the levels of cell-cell fusion without affecting cell surface expression and ephrinB2 binding levels.

**FIG 2:**
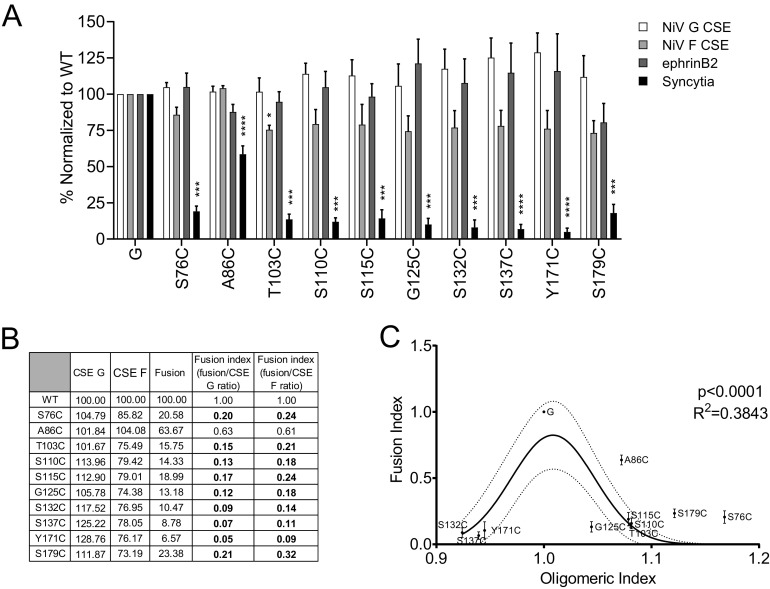
NiV G mutants yielded decreased cell-cell fusion levels. (A) CSE levels of NiV G and F along with ephrinB2 binding levels, determined by flow cytometry. Fusion promotion capabilities (syncytium formation) of wild-type NiV G and cysteine mutants cotransfected with wild-type NiV F. Error bars indicate the standard error of the mean. Averages from three or more independent experiments are shown. (B) CSE and cell-cell fusion levels of NiV G stalk mutants were normalized to those of wild-type NiV G and set to 100%. The ratios of the normalized fusion to G or F cell surface expression (fusion indices) were determined. Values shown in bold had a >4-fold increase in levels of hypofusogenic phenotypes at 16 hpt. (C) Plot of the fusion index from panel B versus oligomeric index from Fig. 1E. The Gaussian distribution curve fit values were determined in GraphPad Prism.

We then assessed whether the NiV G Cys mutants affected protein function via a cell-cell fusion (syncytium formation) assay. We cotransfected a wild-type NiV F construct along with wild-type or mutant NiV G constructs into HEK293T cells. At 16 h post-transfection (hpt), we measured the levels of cell-cell fusion produced by counting nuclei inside syncytia at a ×200 magnification, as we previously established (19, 29, 34, 35). We determined that all of the cysteine mutants had a significant decrease in syncytium formation (5 to 25% of wild-type G levels), except for mutant A86C, which showed only a mild effect on syncytium formation (~63% of wild-type levels) (Fig. 2A). Since syncytium formation is highly dependent on cell surface expression (CSE) levels of the glycoproteins, we determined the CSE levels of the mutant glycoproteins via flow cytometry, using an antibody that binds the extracellular HA tag of G and the polyclonal 835 antibody that binds to NiV F (Fig. 2A) (29, 30). The NiV G cysteine mutant CSE levels were normalized to that of wild-type NiV G, set to 100%. All mutants exhibited CSE levels that were not significantly different compared to those of wild-type NiV G. Although the S137C and Y171C mutants appear to have slightly elevated levels of CSE, when statistically analyzed (*P* values of 0.1591 and 0.1229, respectively), there was no significant statistical difference. Similarly, the levels of NiV F CSE were not statistically different from that of F in the wild-type G/F pair, except for a mild decrease in F CSE for the T103C mutant (*P* < 0.05). The F CSE data for the G125C and S179C mutants show slightly decreased levels as well; however, when statistically analyzed, there was no significant statistical difference (*P* values of 0.1378 and 0.0879, respectively). This difference in expression has been observed previously and may be caused by the cell’s limitations in effectively producing both glycoproteins (32). Therefore, the G or F CSE levels were not likely a significant cause for the very low syncytia levels observed for most G mutants. To be more certain of this conclusion, we determined a fusion index via the calculation of the ratio of the normalized fusion levels to normalized CSE levels, as we previously performed in several of our studies (29, 31, 35, 36). The NiV G CSE or F CSE (from F/G-transfected cells) and cell-cell fusion levels were within the previously determined linear ranges for these assays (29, 36). We observed that nearly all of the cysteine mutants displayed hypofusogenic phenotypes regardless of whether we used CSE values from G or F, yielding fusion indexes between 0.05 and 0.32, except for the A86C mutant, which yielded fusion indexes of 0.61 and 0.63, more similar to that of wild-type NiV G (Fig. 2B). Subsequent analyses were performed using the fusion index for G CSE (fusion/CSE G).

Although all cysteines were introduced into the stalk domain, since syncytium formation also depends on NiV G binding to cell receptors, the levels of ephrinB2 receptor binding of the NiV G cysteine mutants were analyzed via flow cytometry using a soluble mouse ephrinB2/human Fc chimeric protein, as previously established (16, 31, 32, 37). All NiV G cysteine mutants bound to soluble ephrinB2 at levels relatively similar to that of wild-type NiV G (Fig. 2A). Together, these data indicate that the significant reduction in cell-cell fusion observed for most of the cysteine mutants was not due to altered levels of glycoprotein cellular expression, transport to the cell surface, degradation, or binding to the cell receptor. These results strongly suggest that increased tetrameric strength propensity; in other words, restriction of the mobility of the G tetrameric stalk structure at various points in its length is linked to G’s lower intrinsic capacity to promote membrane fusion. That is, the freedom of mobility of the G stalk may be required to efficiently promote membrane fusion. Interestingly, we observed that the mutants with either much higher or lower oligomeric indexes compared to that of wild-type NiV G showed a steep decrease in fusogenicity, while A86C with a similar oligomeric index to that of wild-type G had similar fusogenicity levels (Fig. 2C). This suggests there is an important balance of the G oligomeric states that must be maintained for optimal levels of membrane fusion promotion.

### Hypofusogenic NiV G stalk cysteine mutants may prematurely expose the G stalk.

**FIG 3:**
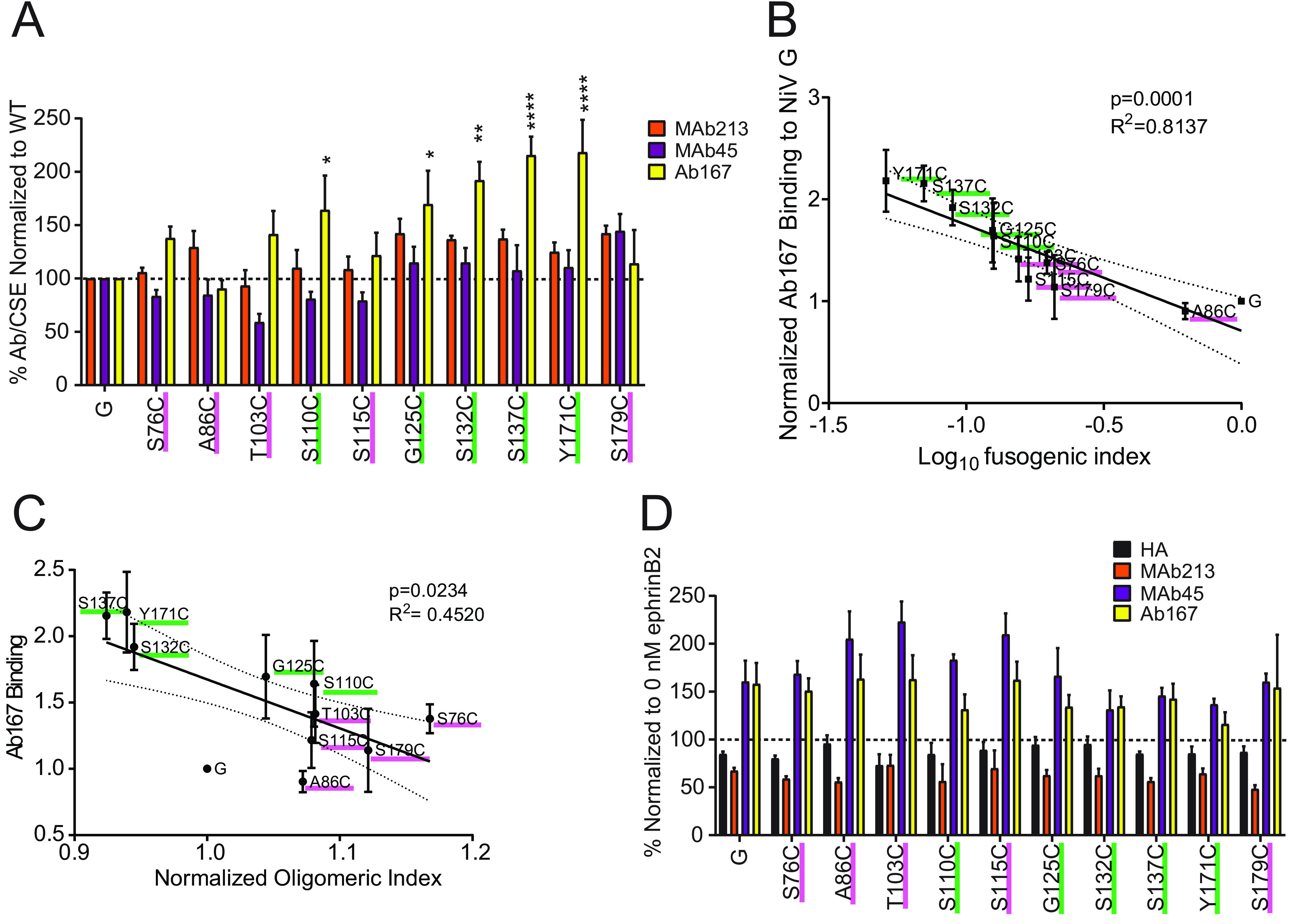
NiV G stalk mutants prematurely expose the G stalk and quantitatively alter receptor-induced conformational changes. (A) EphrinB2-deficient PK13 cells were transfected with either wild-type NiV G or cysteine mutants. Levels of CSE measured with the anti-HA tag, and binding values of MAb26, MAb45, MAb213, and Ab167 were determined through flow cytometry and normalized to those of wild-type NiV G. (B) Ab167 binding values from panel A plotted against the fusion index (fusion/CSE G) scores from Fig. 2B. Pearson correlation analysis was performed using GraphPad Prism. (C) Values of Ab167 binding to G from panel A plotted against the oligomeric indexes from Fig. 1E. Pearson correlation analysis was performed using GraphPad Prism (D) NiV G mutants binding to MAb26, MAb45, MAb213, and Ab167 in either the absence (0 nM) or presence (100 nM) of soluble ephrinB2. Binding levels determined in the presence of ephrinB2 were normalized to those in the absence of ephrinB2. Two-way ANOVA was performed, followed by Dunnett’s test. Error bars indicate the standard error of the mean. Four or more independent experiments are shown.

To determine whether gross conformational changes in NiV G were caused by the introduction of cysteines to the stalk domain, we used a panel of previously established conformational monoclonal antibodies (MAbs) with NiV G epitopes (16, 17, 38). We previously reported three post-receptor-induced conformational changes in NiV G that can be detected using three conformational antibodies. MAb213 measures the first conformational change in head region 9 (residues 371 to 392), MAb45 measures the second conformational change in head region 4 (residues 195 to 211), and Ab167 measures the third conformational change, the exposure of a stalk region (residues 159 to 167) (16, 38, 39). We observed no significant changes in the gross native conformations of the NiV G head for any of the cysteine mutants (Fig. 3A), assessed by binding of anti-head epitope MAb213 and MAb45. Although there are slight apparent reductions in MAb45 binding to S76C, T103C, S110C, and S115C, when statistically analyzed (*P* values of 0.9922, 0.4670, 0.9834, and 0.9709, respectively) the levels of binding were not significantly statistically different from that of the wild type. Mutants A86C, G125C, S132C, S137C, Y171C, and S179C appear to have slight increases in MAb213 binding. However, when statistically scored (*P* values of 0.7258, 0.3081, 0.4741, 0.4502, 0.8603, and 0.3101, respectively), binding is not statistically significantly different from that of wild-type NiV G. However, some of the most hypofusogenic mutants appeared to also display increased binding to Ab167 (mutants S110C, G125C, S132C, S137C, and Y171C), which signifies a relative increased exposure of a stalk region (16, 17, 32). Interestingly, when we plotted the Ab167 binding levels in Fig. 3A against G fusion index levels in Fig. 2B, we observed a significant inverse correlation (*P* = 0.0001) between Ab167 binding and fusion (Fig. 3B). To further address whether this premature G stalk exposure phenotype correlated with oligomeric strength, we then plotted the normalized oligomeric indexes from Fig. 1E versus Ab167 binding to G in Fig. 3A. We observed a mild but significant inverse correlation between relative oligomeric strength and Ab167 binding (Fig. 3C). Together, these data suggest that prior to receptor binding, relative exposure of the Ab167 G stalk epitope(s) negatively correlates with both oligomeric strength and fusogenic capacity.

Furthermore, receptor-induced antibody binding phenotypes were measured and then normalized to wild-type NiV G (set at 100% in the absence of receptor ephrinB2). Our results indicate that as wild-type NiV G, all of the NiV G stalk mutant glycoproteins were able to undergo all three receptor-induced conformational changes (Fig. 3D). However, we noticed that the mutants that displayed higher levels of Ab167 binding prior to receptor binding (green underline) in Fig. 3A appeared to display relatively lower levels of Ab167 binding following receptor binding (pink underline) (Fig. 3D). We thus calculated the ratio of the levels of Ab167 binding to G after ephrinB2 receptor binding (Fig. 3D) over the levels of Ab167 binding to G prior to ephrinB2 receptor binding (Fig. 3A).

### NiV G stalk mobility is key to F triggering.

**FIG 4:**
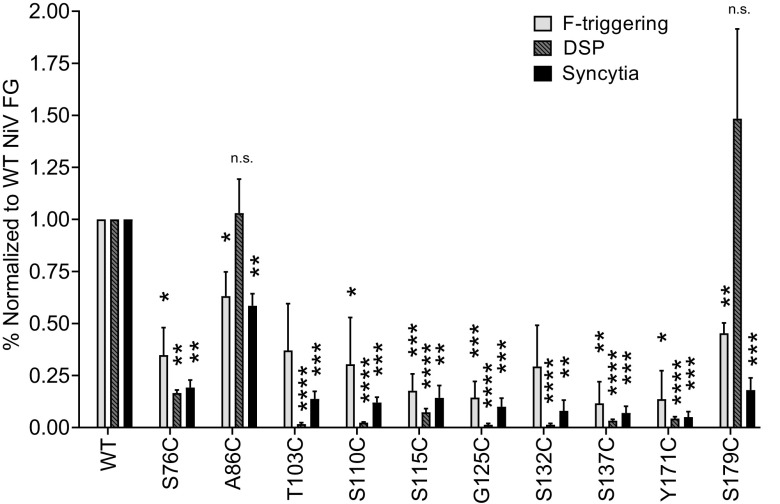
The NiV G C-terminal region has important roles in F triggering and extensive fusion pore expansion. F-triggering levels were determined through flow cytometry at 12 hpt, at which point low levels of syncytia were observed for wild-type G/F and were normalized to those of wild-type NiV G and F. HEK293T effector cells were transfected with DSP1, wild-type NiV F, and wild-type or mutant NiV G. Target cells were transfected with ephrinB2 and DSP2. At 12 hpt, cells were harvested and incubated in the presence of EnduRen substrate and scanned for luminescence every 2 h on a Tecan plate reader. Data are shown at 4 h post cell mixing. Syncytium values from Fig. 2A are shown as well for the convenience of the reader.

We reasoned that although all three receptor-induced conformational changes occur for all mutants (Fig. 3D), the relatively premature exposure of the stalk prior to ephrinB2 exposure (Fig. 3A, B) may result in less effective fusion, perhaps by affecting F triggering, which was further analyzed using F-triggering assays (19). After receptor binding, conformational changes in the head of G expose the stalk, which in turn triggers F to shift from the metastable prefusion conformation into the prehairpin intermediate (PHI), a step we define as F triggering. Our lab has developed an F-triggering assay that detects formation of the PHI by utilizing a soluble fluorescently labeled HR2 peptide (Cy5-HR2) that mimics F’s native HR2 region. As F transitions to the PHI, the HR1 domain is exposed, thus allowing Cy5-HR2 to bind and be detected by flow cytometric analysis (35, 38, 40). Notably, most cysteine mutants yielded reduced levels of F triggering compared to that of wild-type NiV G, again with the A86C mutant yielding the levels most similar to those of wild-type NiV G (Fig. 4). Importantly, such results are generally consistent with the corresponding observed decreases in syncytium formation (Fig. 4). These data suggest that a relative oligomeric constrain and potential partial premature exposure of the stalk prior to receptor binding correlates with less effective triggering of the F protein, a novel mechanistic finding for the Paramyxoviridae family.

### Cysteine mutant S179C triggers NiV F and allows fusion pore formation but hinders fusion pore expansion.

To determine whether the cysteine substitution mutants along the NiV G stalk were capable of a downstream step, forming small membrane fusion pores that are sufficient in size for cytoplasmic protein-protein mixing, we completed a dual split protein (DSP) Renilla luciferase-green fluorescent protein kinetic assay. This assay measures cytoplasm mixing between two cells upon fusion pore formation in real time, and we have recently adapted it to NiV G/F (35, 41–45). As expected, we found that for most hypofusogenic mutants, the ability to form fusion pores was severely decreased. We reason that the nearly complete lack of DSP signal for some of the most hypofusogenic mutants is likely due to a lack of signal detection sensitivity in this assay relative to the levels in the negative control (G only) subtracted from these signals, in contrast to the low levels of syncytia microscopically visible for such mutants. Again, consistently with prior results, the level of DSP signal was less affected for mutant A86C than for wild-type NiV G, as expected. Most interestingly, we observed higher levels of fusion pore formation for the S179C mutant (Fig. 4 and Table 1). Interestingly, however, the S179C mutant displayed low syncytium levels with a fusion index of 0.21. These results indicate that a step occurring after fusion pore formation but prior to full syncytium formation, such as fusion pore expansion, is significantly decreased by this mutant. We posit that the S179C mutant allows the formation of fusion pores sufficiently large to allow cytoplasmic mixing. However, since the S179C mutant yielded only low levels of syncytium formation, the pore formation promoted by S179C must not expand extensively enough for the exchange and gathering of whole large organelles, such as nuclei, within the cells. Thus, the S179C mutant appears to inhibit extensive fusion pore expansion. As far as we are aware, this phenotype has not been observed for any paramyxoviral RBP mutant. Thus far, the paramyxoviral HN, H, or G have been ascribed two essential roles, host cell receptor binding and F triggering. These data suggest, however, that residues within the stalk domain may be important for regulating not only tetrameric strength and F triggering, but also a post-F-triggering step or steps in the fusion cascade.

**TABLE 1:** Summary of steps in the fusion cascade affected by G cysteine mutants

Sample	Levels^*a*^									Fusion step affected
	CSE G	Monomer formation	Tetramer/dimer ratio	Ab167 binding prior to receptor exposure	EphrinB2 binding	F triggering	Fusion pore formation	G-F avidity	Syncytia	
WT	++	++	++	++	++	++	++	++	++	Normal
S76C	++	+	++	+++	++	+	+	++++	+	F triggering
A86C	++	++	++	++	++	++	++	++	++	Nearly normal
T103C	++	++	+++	+++	++	+	−	++++	−	F triggering
S110C	++	+++	++++	++++	++	+	−	++++	−	F triggering
S115C	++	++	+++	+++	++	−	−	++++	−	F triggering
G125C	++	+++	++++	++++	++	−	−	++++	−	F triggering
S132C	++	++++	++	++++	++	−	−	++++	−	F triggering
S137C	++	++++	++	++++	++	−	−	++++	−	F triggering
Y171C	++	++++	++++	++++	++	−	−	++++	−	F triggering
S179C	++	++	++++	++	++	+	++	++++	+	Fusion pore expansion

a −, Near negligible; +, lower than wild type; ++, wild type or near wild type; +++, slightly higher than wild type, but not statistically significant; ++++, statistically significantly higher than wild type.

**FIG 5:**
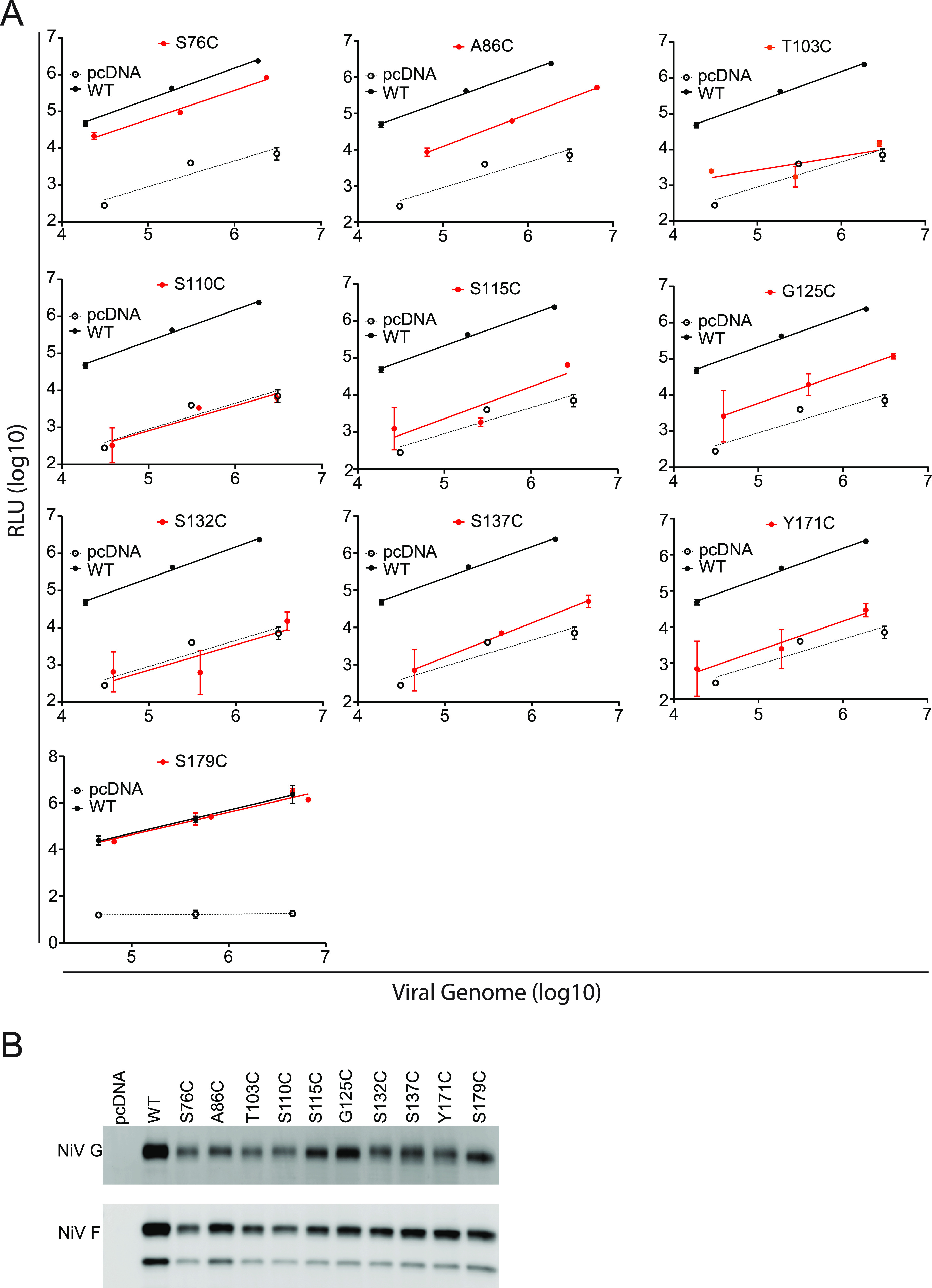
Viral entry of NiV/VSV pseudotyped virions with wild-type NiV F and either wild-type NiV G or mutant proteins. (A) Cells were lysed at 24 h post infection (hpi) and relative light units (RLU) measured. Values for mutant VSV virions were compared to those of wild-type NiV G/F virions and to the negative-control bald pseudotyped virions produced using only the pcDNA3.1^+^ expression plasmid. The average and standard deviation (SD) of 3 experiments is shown. (B) Western blot analysis of the virions analyzed. One representative experiment is shown.

We next sought to determine whether the cysteine mutant syncytium and viral entry phenotypes were similar. Thus, we used our previously established pseudotyped virus particle entry assay (Fig. 5A) (29–31, 35, 46). Vesicular stomatitis virus (VSV) with a *Renilla* luciferase reporter gene in place of the natural VSV G (VSV-ΔG-rLuc) was pseudotyped with wild-type NiV F and either wild-type NiV G or mutant NiV G proteins. To quantify and fairly compare the relative viral entry levels of the wild type and the cysteine mutants, the pseudotyped VSV genome copy numbers were determined through quantitative reverse transcription-PCR (RT-qPCR). The amounts of wild-type or mutant NiV G/F were then normalized to equivalent viral genome copy numbers to prior to cell infection. In addition, equal numbers of genome copies (roughly equivalent to equal numbers of virions) were subjected to Western blot analysis to account for major discrepancies in glycoprotein incorporation, as previously established (29–31, 35, 46). Infected cells were lysed, and luciferase activity was measured. Generally, the viral entry levels correlated well with the cell-cell fusion levels for the cysteine mutants. The T103C, S110C, S115C, G125C, S132C, S137C, and Y171C mutants had viral entry levels similar to that of the negative pcDNA 3.1^+^ virus. The A86C mutant viral entry levels were closer to that of wild-type NiV G virions. One exception was the S76C mutant, for which the VSV pseudotyped virions entered cells at levels similar to wild-type levels despite having a hypofusogenic cell-cell fusion phenotype. Notably, the levels of viral entry for the S179C mutant were similar to those of wild-type NiV G virions, correlating with the fusion pore formation levels, but not with the syncytium levels observed for mutant S179C (Fig. 5A). Interestingly, the levels of incorporation of the cysteine proteins were generally lower than those for wild-type NiV F and G, which may account for some of the lack of viral entry for some of the cysteine mutants (Fig. 5B). The findings that the levels of viral entry and syncytium formation are not always equivalent has been previously observed (31, 35, 36, 46, 47). We propose that the S179C mutant can effectively promote the formation of fusion pores sufficiently large to allow protein mixing (in our DSP assay), as well as viral genome entry into cells, but not extensive enough to allow nuclei movement and gathering during the process of cell-cell fusion. These results ascribe to G or G/F interactions a novel role(s) late in the membrane fusion cascade, such as in the extensive fusion pore expansion needed for syncytium formation.

### Hypofusogenic mutants have increased avidities of G/F interactions.

There are several suggested models by which the two paramyxoviral surface glycoproteins interact and promote membrane fusion. In short, in variations of the dissociation models, the two glycoproteins interact prior to receptor binding, and upon receptor binding, relative G/F dissociation and triggering of the fusion glycoprotein occur. In variations of the association models, the attachment and fusion glycoproteins interact relatively more strongly upon receptor binding to allow for fusion glycoprotein triggering (12, 30, 48, 49). Regardless of the model, it is well accepted that attachment and fusion glycoprotein interactions are key to modulating the membrane fusion process for the paramyxoviruses (5, 21, 49).

**FIG 6:**
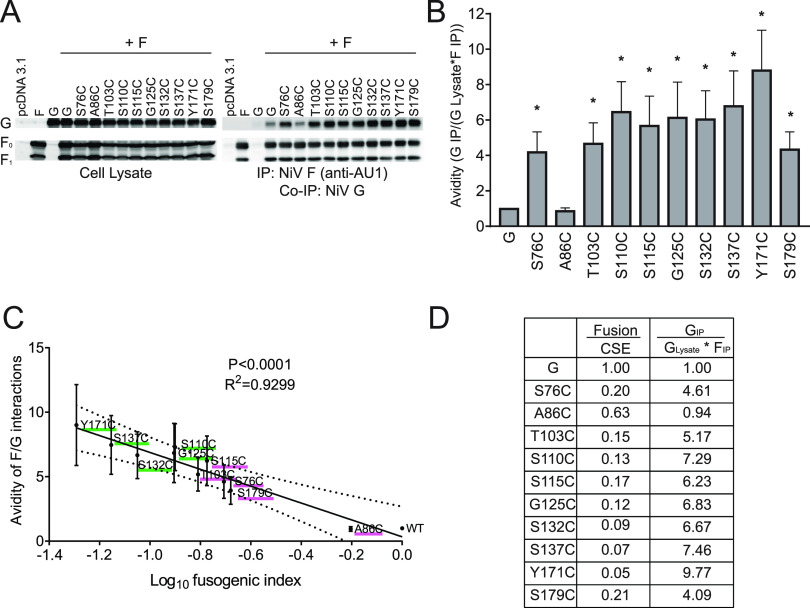
Mutants that reduce cell-cell fusion yield increased avidities of G/F interactions. (A) Representative Western blot image of the NiV G and F interactions determined for wild-type NiV G or cysteine mutants by immunoprecipitating NiV F using protein G microbeads incubated with an antibody against the AU1 tag. Cell lysates and coimmunoprecipitated proteins (Co-IP; anti-NiV G) were run on a 10% SDS-PAGE gel. (B) The ratio of the coimmunoprecipitated G to the total cell lysate of G * F IP was determined by fluorescence densitometry, measured using Bio-Rad ImageLab software. (C) Avidities of the G/F interactions from panel B were plotted against the fusion values from panel B. Pearson correlation analysis was determined using GraphPad Prism. (D) Table showing the fusion indexes (fusion/CSE G) from Fig. 2B and the avidities of G/F interactions from panel B.

Our prior studies for various NiV G and F mutants have shown a typical negative correlation between the avidity of G/F interactions and the fusogenic capacity of most NiV G or F mutants, supporting variations of the dissociation models with various levels of complexity (29–31). To determine whether the NiV G cysteine mutants altered the avidity of NiV G-F interactions, we completed coimmunoprecipitation assays by cotransfecting HEK293T cells with NiV G and F. The affinity purification was against the NiV F C-terminal AU1 tag so that only the G proteins associated with F would be pulled down. Importantly, these tagged proteins have been shown to be fully functional (29, 30). The surface glycoproteins were then detected from the total cell lysates or the immunoprecipitated eluate via immunoblotting using either rabbit anti-HA (NiV G) or mouse anti-AU1 (NiV F) (Fig. 6A) antibodies, as we previously established (35, 47). Interestingly, we observed a significant 4- to 9-fold increase in the avidities of G/F interactions for the hypofusogenic G stalk mutants, while the A86C mutant, which yielded near-wild-type levels of syncytium formation, also yielded a near-wild-type avidity of G/F interactions (Fig. 6B). Further, compared to wild-type NiV G, mutants with higher tetrameric strength (G125C, S132C, S137C, and Y171C) also displayed high Ab167 binding prior to receptor exposure, high levels of F/G avidity, and low levels of F triggering and subsequent fusion pore formation (Table 1). Furthermore, when we plotted the relative avidity of G/F interactions for wild-type NiV G versus cysteine mutants against their respective fusion indexes from Fig. 2B, we obtained a significant negative correlation (Fig. 6C and D, *r*^2^ = 0.9299, *P* < 0.0001). These data suggest that the most hypofusogenic cysteine mutants have higher avidities of G/F interactions, supporting the notion that the G stalk and its mobility (oligomeric looseness) correlate with relative looseness of the G/F interactions, which may be important for effective F protein triggering and subsequent steps during the membrane fusion process.

## DISCUSSION

**FIG 7:**
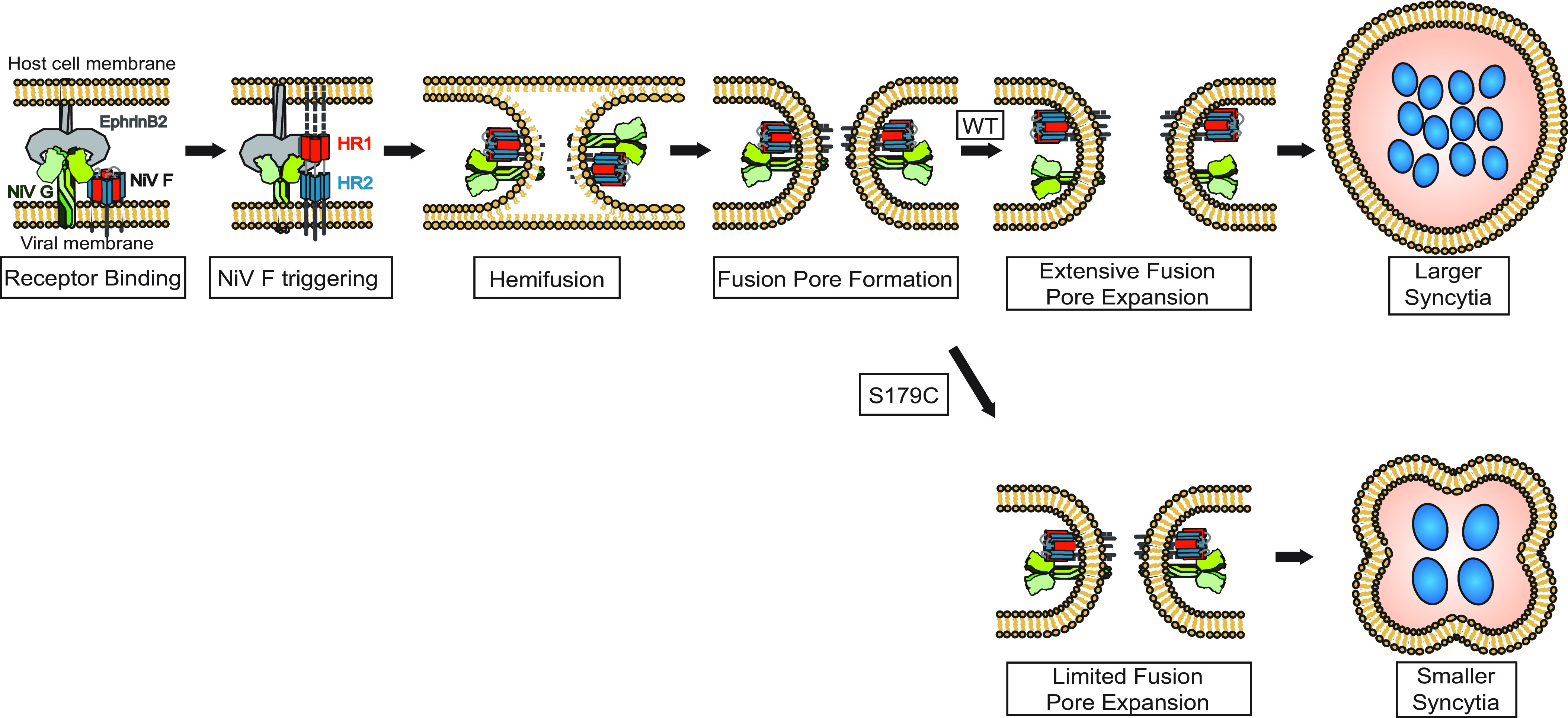
A new model of NiV membrane fusion. NiV G and F surface glycoproteins expressed on the viral membrane. NiV G binding to receptor ephrinB2 or B3 results in F triggering, with the formation of the prehairpin intermediate (PHI), whereby NiV F HR1 (red) and HR2 (blue) regions have a high affinity for one another and come together to form 6-helix bundles (6HB), promoting hemifusion, followed by fusion pore formation and fusion pore expansion to complete viral-cell or cell-cell membrane fusion. In addition, for the S179C mutant, the interactions between F and G remain stronger, leading to limited fusion pore expansion and lower observed levels of cell-cell fusion.

Paramyxoviral fusion with the host cell membrane is a prerequisite for viral entry, subsequent cell infection, and syncytium formation. To accomplish this, paramyxoviruses use the coordinated efforts of their two glycoproteins (49–51). We and others have reported the stalk domains of paramyxoviral attachment glycoproteins playing significant roles in their interactions with and triggering of the F glycoprotein (16, 27, 32, 38, 52–55). However, how those interactions influence the fusion cascade, particularly at its late stages, has remained a critical knowledge gap. Current henipaviral models suggest that NiV G plays at least two key roles in viral entry. The first is to bind to the host cell receptor and undergo sequential conformational changes that expose the G stalk’s C-terminal domain. At this point, the second role of G is to trigger F (Fig. 7A and B) (16, 17, 32). Henipaviral fusion models suggest that the G and F glycoproteins dissociate at some point to allow the fusion cascade to proceed. However, how G/F interactions link receptor binding to F triggering and potentially to steps beyond F triggering remains a critical knowledge gap not only for the henipaviruses but more generally for the paramyxoviruses. Here, we identified new roles for G stalk mobility and/or G/F dissociation in F triggering and extensive fusion pore expansion.

There are a few different models for how paramyxoviral G can send the signal to F and initiate membrane fusion activation. The sliding model proposes that after host receptor binding, the attachment glycoprotein can physically slide laterally to a staggered configuration while the attachment glycoprotein stalk domain partially dissociates to release itself from the F glycoprotein (56, 57). The stalk exposure model proposes that host receptor binding induces conformational changes in the attachment glycoprotein globular head from the “heads-down” to a “heads-up” conformation. Once at the heads-up conformation, the stalk domain becomes exposed and can interact with F (16, 58–60). Interactions that occur between NiV F and G glycoproteins prior to host receptor binding led to the proposed bidentate model. This model proposes that the G head and stalk domains directly interact with F prior to receptor binding, after which there is a conformational shift to favor interaction of the G stalk domain and F (30, 32, 38, 61). The oligomerization model proposes that receptor binding increases multimerization of attachment glycoprotein, which triggers F. This model was suggested for HPIV-3 due to the conformational changes in the HN globular head not being sufficient to trigger F (62). In addition, the clustering of host receptors has also been proposed to aid attachment protein multimerization to activate the prefusion F (63). Importantly, these models are not mutually exclusive and can coexist for given paramyxoviruses.

A recent study reported that the rigidity of the MeV H head-stalk linker region can alter the tetramerization of H and subsequent membrane fusion promotion (33). Here, we introduced cysteine mutations along the entire NiV G stalk domain from the N terminus to the C terminus (Fig. 1A) to determine whether its altered oligomeric structure would influence G/F interactions, F triggering, or later fusion events such as fusion pore formation and expansion. Generally, these cysteine mutants formed NiV G oligomeric structures with higher tetramer-forming propensities compared to that of wild-type NiV G. Importantly, the mutants were capable of binding to the host cell receptor ephrinB2 and undergoing conformational changes similar to those of wild-type NiV G, and thus these were not the reasons for the mutants’ hypofusogenic phenotypes. Interestingly, the S110C, G125C, S132C, S137C, and Y171C mutants yielded significantly increased levels of Ab167 binding, suggesting that at least one epitope in the stalk domain is relatively more exposed in those mutants than in wild-type NiV G prior to receptor binding. Importantly, this relative exposure of the stalk before receptor binding yielded lower stalk epitope exposure post receptor binding, and this appears to be deleterious to the membrane fusion process, as it results in reduced levels of F triggering. A possible explanation may be that G potentially undergoes its own unforeseen spring-loaded-like mechanism of activation, whereby when a G mutant is already in a relatively more advanced post-receptor-activated G conformation, the energy that G provides for F triggering will be lower, or not as effective to trigger F. Current hypothetical models for paramyxovirus G/F interaction have suggested that upon receptor binding, G triggers F to undergo its own F spring-loaded mechanism, which results in membrane fusion (18, 35, 51, 64). An interesting observation from Fig. 1D and E was that some mutants had higher levels of both tetramers and monomers, while others had an increase in only tetramer or only monomer levels, pointing to increased conformational heterogeneity for some of the mutants, roughly corresponding to an increase in Ab167 binding prior to ephrinB2 binding. We posit that this increased conformational heterogeneity may allow for the globular head to shift and move away to expose NiV G stalk epitopes to Ab167. This in turn may lead to a premature presentation of the G stalk that results in lower levels of F triggering. Another possibility is that this exposure of the G-stalk domain places the protein in a conformation that is suboptimal for F triggering. Further research is needed to determine the mechanistic underpinnings of how prereceptor G-stalk exposure may result in lower levels of F triggering and subsequent syncytium formation. It is noteworthy that this discovery may have repercussions for activation of other paramyxoviral G/H/HN glycoproteins and their fusion processes.

The coimmunoprecipitation assays revealed that most cysteine mutants were capable of interacting with NiV F at avidity levels significantly higher than those of the wild type. Typically for the henipaviruses, the binding avidity of G and F mutants inversely correlates with their respective levels of fusogenicity, as we previously reported (29–31, 47). These inverse correlations support the G/F dissociation models, whereby the interactions between G and F must decrease so the membrane fusion cascade may proceed. Interestingly, most of the cysteine mutants, except A86C, yielded high levels of G/F avidities, up to a 9-fold increase compared to that of wild-type NiV G, implicating the stalk and its mobility in such a dissociation/fusion relationship (Fig. 3B). In addition to the higher avidity levels, the location of the cysteine mutant appears to also play a role. To illustrate this point, the S115C mutant displays a similar G/F avidity level as the S179C mutant (Fig. 6), however, the F-triggering capacity of mutant S115C is much lower than that of mutant S179C (Fig. 4). This suggests that F is capable of being triggered even when having a relatively strong interaction with G, depending on the position in the G stalk that affects such G/F interactions. This gives insight into the potential role(s) G and/or G/F interactions play post receptor binding.

Mutant S179C was successful in effectively promoting F triggering and fusion pore formation, but not syncytium formation. This points to a role of G/F interactions late in the fusion cascade in extensive fusion pore expansion, an unforeseen phenotype in the paramyxoviral membrane fusion field. Based on our findings, we offer a model in which G and F interactions or their dissociations are important beyond F triggering. We propose that G (or G/F interactions) has at least one additional role beyond receptor binding and F triggering, as late as in extensive fusion pore expansion, an unprecedented result (Fig. 7). However, it is likely not solely the dissociation of G and F that allows extensive fusion pore expansion, since other stalk cysteine mutants that displayed similarly increased levels of G/F interaction avidities to that of the S179C mutant did not affect extensive fusion pore expansion. Our model suggests that rather than dissociating from F early in the fusion cascade, G may continue its interaction with F until late points in the fusion cascade, although such interactions need to be sensitively modulated (Fig. 7). Our model is supported by data from human parainfluenza virus type 3 (HPIV-3) and chimeric NiV/HPIV-3 attachment glycoprotein studies, which suggests the involvement of receptor engagement to allow fusion glycoprotein to remain activated (25, 26, 65). Here, we analyzed individual locations in the stalk domain important for G/F interaction(s) and dissected their roles in various steps of the membrane fusion cascade. We propose that G/F interactions may have distinct stages of association and dissociation, perhaps as late as during extensive fusion pore expansion. Further research may elucidate precisely how G/F interactions differ at each step of the membrane fusion cascade.

## MATERIALS AND METHODS

### Cell lines.

Human embryonic kidney (HEK293T), porcine kidney (PK13), and African green monkey kidney (Vero) cell lines (ATCC, Manassas, VA) were cultured with Dulbecco’s modified Eagle’s medium (DMEM) supplemented with 10% fetal bovine serum (FBS), 1% penicillin/streptomycin, and 1% HEPES at 37°C with 5% CO_2_ (66).

### Expression plasmids and site-directed mutagenesis.

NiV envelope nucleotide sequences of NiV F (GenBank accession number AY816748) and NiV G (GenBank accession number AY816746) from the Malaysian strain were codon optimized. The genes were subcloned into the pcDNA 3.1^+^ and pCAGGS vectors, respectively, to improve protein expression (29, 37, 67). The envelope glycoproteins were tagged at their C termini with a hemagglutinin (HA) tag on NiV G and an AU1 tag on NiV F. The NiV G stalk cysteine mutants were constructed by site-directed mutagenesis (Agilent Technologies, Foster City, CA), and all mutations and full genes for each mutant were verified through DNA sequencing (Eurofins Genomics, Louisville, KY).

### Reducing and nonreducing gel electrophoresis and blotting.

HEK293T cells were transfected with wild-type NiV F and either NiV G or G mutant plasmids into 6-well plates. At 24 hpt, cells were harvested and lysed using 1× radioimmunoprecipitation assay (RIPA) buffer (Merck Millipore, Temecula, CA) supplemented with cOmplete protease inhibitor tablet (Roche, Indianapolis, IN). Cell lysate samples undergoing nonreducing SDS-PAGE were loaded onto gels without further treatment using reducing-agent-free 1× Laemmli sample buffer as performed previously (31, 47). NiV G and F proteins were detected by incubating overnight at 4°C with anti-HA (1:2,000) or anti-AU1 (1:1,000) antibodies, respectively. Fluorescent secondary antibodies (Alexa Fluor 488 goat anti-mouse or Alexa Fluor 647 goat anti-rabbit at 1:1,000) were incubated for 1 h.

### Ellman’s reagent assay.

Samples for either the cysteine hydrochloride monohydrate standard curve (0.0 to 1.5 mM) or 35 μM NiV G (wild type or cysteine mutants) were prepared in a reaction buffer (100 mM sodium phosphate and 1 mM EDTA at pH 8.0) along with 4 mg/mL Ellman’s reagent (Thermo Fisher, Darmstadt, Germany). We followed the previously described 96-well-plate method and measured absorbance at 412 nm (68).

### Cell-cell fusion assay.

HEK293T cells were transfected using 3 μg of total DNA for NiV F and NiV G or mutant NiV G expression plasmids (1:1 ratio). At 16 to 18 hpt, syncytial nuclei (>4 nuclei per cell) were counted in each microscopic field for a total of five fields at 200× magnification (29, 31).

### Detection of cell surface expression, ephrinB2, and antibody binding using flow cytometry.

HEK293T cells were transfected using 3 μg of NiV G or mutant NiV G plasmid and collected at 16 to 18 hpt.

### (i) Cell surface expression.

Quantification of CSE for wild-type NiV F, wild-type NiV G, and cysteine mutants were performed using rabbit anti-F polyclonal antisera 835 and mouse anti-HA using 1:500 and 1:200 dilutions, respectively, for 1 h at 4°C (29, 35, 66). The bound anti-F antibody was detected using Alexa Fluor 647 goat anti-rabbit antibodies (Bio-Rad, Hercules, CA). Bound anti-HA antibody was detected using Alexa Fluor 488 goat anti-mouse antibodies as previously performed (Bio-Rad) (31, 47).

### (ii) Fusion index calculation.

Flow cytometry was performed on cells expressing both NiV F and G, and from those values, the NiV F or G cell surface expression levels were used along with the fusogenicity values from the cell-cell fusion assay to determine the fusion index.

### (iii) EphrinB2 binding.

The binding of soluble mouse ephrinB2 fused to human Fc (100 nM); R&D Systems, Minneapolis, MN) to NiV G was measured through flow cytometry. Bound soluble ephrinB2 was detected using a goat anti-human antibody (Caltag, Burlingame, CA).

### (iv) Antibody binding.

The NiV-G conformations were measured by detecting binding of anti-NiV G rabbit conformational MAb26, MAb45, or MAb213, or a mouse anti-HA control, as shown previously (16, 36). A 1:100 dilution was used for primary antibodies and Alexa Fluor 647 goat anti-rabbit or Alexa Fluor 647 goat anti-mouse secondary antibodies.

### (v) PK13 conformational Ab binding.

Flow cytometry assays using rabbit anti-NiV G monoclonal antibodies MAb26, MAb45, MAb213, polyclonal Ab167, and HA Ab were completed as previously described (16, 36). The cells were transfected with Lipofectamine 2000 following the manufacturer’s protocol and harvested at 24 hpt. Cells were incubated for 15 min at 4°C with 0 nM or 100 nM soluble ephrinB2 and subsequently incubated for 1 h at 37°C with MAb26, MAb45, MAb213, Ab167, and HA (1:1,000). Ab binding was detected using Alexa Fluor 647 (1:200).

### Coimmunoprecipitation.

Equal amounts of F and/or G expression plasmids (total 3 μg) were transfected into HEK293T cells (~90% confluence in 6-well plates, 1 well per sample). At 24 hpt, supernatant and cells were harvested using 1 mL ice-cold 1× phosphate-buffered saline (PBS) per well. Cell and supernatant mixtures were centrifuged at 250 × *g* for 5 min. Cell pellets were lysed on ice for 20 min and vortexed every 5 min using 1× RIPA buffer supplemented with 1× protease inhibitor cOmplete Ultra (Roche), using 200 μL per sample. Cell lysates were then centrifuged at 12,500 × *g* for 20 min at 4°C, and the supernatant was carried forward for analysis. Cell lysate supernatants were separated into two tubes, one half for direct SDS-PAGE analysis and the other for coimmunoprecipitation via a μMACS protein G isolation kit (Miltenyi Biotec, Auburn, CA). The mixture of 30 μL μMACS protein G microbeads with 4 μL of rabbit anti-AU1 antibody were rotated overnight at 4°C. A 34-μL aliquot of the bead/Ab mixture was added to 100 μL of cell lysate and incubated at 4°C for 2.5 h. Tagged proteins bound to the μMACS columns were washed 3 times with 300 μL lysis buffer (25 mM Tris-HCl, 0.15 M NaCl, 1.0 mM EDTA, 1% NP-40, and 5% glycerol) followed by one wash with 100 μL low salt lysis buffer (1% NP-40 and 50 mM Tris-HCl [pH 8.0]). Coimmunoprecipitation eluates and cell lysates were analyzed using 10% acrylamide gels and blotted onto polyvinylidene difluoride (PVDF) membrane.

### Dual split protein cell content mixing assay.

HEK293T cells were seeded into 96-well plates at 6.0 × 10^4^ cells per well. Effector cells were transfected with NiV F and NiV G plasmids, along with the dual split protein (DSP_1–7_) at a 3:1:2 ratio, at ~80% confluence. The DSP_1–7_ and DSP_8–11_ plasmids were a generous gift from Richard K. Plemper (42, 44). Target HEK293T cells were transfected with DSP_8–11_ and ephrinB2 plasmids at a 2:1 ratio for a total of 600 ng DNA. At 12 hpt, cells were washed 4× with 200 μL 1× PBS and centrifuged at 200 × *g* for 5 min. EnduRen live cell substrate (Promega, Madison, WI) was added to each well according to the manufacturer’s instructions. Target cells were lifted, mixed, and incubated with the effector cells at a 1:1 ratio. The mean *Renilla* luciferase activity was determined as relative light units (RLU) and measured using a Tecan Spark plate reader. The experiments were completed in triplicates and repeated independently at least four times.

### F-triggering assay.

F-triggering assays were performed as previously described (38, 47) with some modification. Briefly, HEK293T cells were transfected with a combination of pCAGGS-NiV F and pcDNA 3.1^+^ NiV G (wild type or cysteine mutants) at a 3:1 ratio (1,500 ng:500 ng). Cells were collected before substantial syncytium formation (<50%) and resuspended with chilled 1% bovine serum albumin (BSA) in PBS. Receptor binding occurred at 4°C for 60 min in the presence of 2 μM HR2-Cy5 (Cy5-KVDISSQISSMNQSLQQSKDYIKEAQRLLDTVNPSL) peptide. Subsequently, cells were placed at 37°C to allow F triggering for 30 min. Cells were washed and analyzed through flow cytometric analysis to detect bound HR2-Cy5.

### Pseudotyped NiV/VSV-ΔG rLuc synthesis, RT-qPCR, and viral infectivity/entry assays.

**(i) NiV/VSV ΔG rLuc synthesis.** NiV pseudotyped virions were made using the VSV-ΔG-rLuc virus, namely, the VSV Indiana serotype full-length cDNA clone in which the G glycoprotein was exchanged for the *Renilla* luciferase gene. Two 15-cm^2^ dishes of HEK293T cells were transfected with NiV F and wild-type/mutant NiV G at a 1:1 ratio of 3 μg total DNA and allowed to incubate for 24 h after following the procedures for polyethyleneimine (PEI) transfection. At 12 hpt, VSV G expressing VSV-ΔG-rLuc pseudotyped virions were used to infect the transfected HEK293T cells. Medium was harvested after 48 hpt and ultracentrifuged for 1.5 h at 110,000 × *g* in a 20% sucrose cushion in NTE buffer (150 mM NaCl, 40 mM Tris-HCl, and 1 mM EDTA [pH 8.0]). For viral resuspension, NTE buffer was supplemented with 5% sucrose, and viral aliquots were stored at −80°C.

**(ii) RT-qPCR quantification of viral genomes.** Viral RNA was extracted using the E.Z.N.A. viral RNA kit (Norgross, GA) following the manufacturer’s specifications. The VSV genome copy numbers were determined using quantitative reverse transcription-PCR with UltraPlex 1-Step ToughMix (Quantabio, Beverly, MA) following the manufacturer’s specifications. The TaqMan probe targeting the L gene of the VSV Indiana strain was used at 2.5 μM as described previously (31, 37, 47).

**(iii) Viral entry assay.** Vero cells were seeded at 1.2 × 10^4^ cells per well in a 96-well plate. Cells were infected at ~50% confluence using 10-fold serial virus dilutions. All cells were infected using infectious buffer (1× PBS and 1% FBS) for 2 h, after which the medium was replaced with complete DMEM medium. At 24 h post infection (hpi), medium was removed, and cells were lysed. The *Renilla* luciferase activity was measured as relative light units (RLU) using a *Renilla* luciferase assay system (Promega) and a Tecan Spark microplate reader (Tecan Group, Ltd., Männedorf, Switzerland). The analysis was completed using GraphPad Prism 8.
